# A critical perspective on the modified personal interview

**DOI:** 10.1007/s40037-018-0477-y

**Published:** 2018-10-23

**Authors:** Dilshan Pieris

**Affiliations:** Faculty of Health Sciences, McMaster University, Hamilton, ON Canada

**Keywords:** Admissions, Interviewing, Competencies, Validity

## Abstract

Medical school interviews are critical for screening candidates for admission. Traditionally, the panel format is used for this process, although its drastically low reliabilities sparked the creation of the highly reliable multiple mini-interview (MMI). However, the multiple mini-interview’s feasibility issues made it unappealing to some institutions, like the University of Toronto, who created the modified personal interview (MPI) as a more feasible alternative. The lack of literature about the MPI, however, prevents the medical community from determining whether this interview format achieves this goal. Therefore, evidence was compiled and critically appraised for the MPI using Kane’s validity framework, which enables analysis of four levels of inference (Scoring, Generalization, Extrapolation, Implication). Upon examining each level, it was concluded that assumptions made at the ‘Scoring’ and ‘Generalization’ levels had the least support. Based on these findings, it was recommended that in-person rater training become mandatory and the number of stations increase twofold from four to eight. Moreover, the following research initiatives were suggested to improve understanding of and evidence for the modified personal interview: (1) formally blueprint each station; (2) conduct predictive validity studies for the modified personal interview, and (3) relate admission to medical school on the basis of the MPI with medical error rates. By making these changes and studying these initiatives, the MPI can become a more feasible and equally effective alternative to the MMI with more evidence to justify its implementation at other medical schools.

## Introduction

Interviews are considered the most important factor for selecting medical school candidates [1]. However, there are ongoing debates over which format most effectively selects candidates who will succeed in medicine [1, 2]. Therefore, many schools use either panel interviews or multiple mini-interviews (MMIs) [3].

Panel interviews involve multiple interviewers evaluating a candidate during a single observation [4, 5]. As such, they suffer from context specificity, which is when situational factors affect candidates’ performance more than their true ability [2, 6]. This contributes to the panel interview’s poor reliability—ability to consistently distinguish between applicants of differing levels—of 0.36, which is far below accepted standards (0.70–0.90) [5, 6]. MMIs overcome context specificity by giving candidates 10 opportunities (i. e. stations) to demonstrate their abilities; each station is independently assessed and involves either standardized patients or traditional panel interview questions [2, 5]. Consequently, the MMI’s reliability is between 0.65 and 0.81, exceeding that of the panel interview and falling within acceptable limits [2, 5, 7]. Unfortunately, the MMI, although effective, has logistical issues (availability and cost of a large number of rooms and standardized patients, scenario development) that deter some schools from adopting it [5, 8–10].

The University of Toronto is one school that decided against using the MMI due to its logistical constraints. As such, University of Toronto created the Modified Personal Interview (MPI) to achieve the MMI’s effectiveness with the panel interview’s feasibility [5]. Like the MMI, the MPI attempts to address context specificity by offering four independently assessed stations [5, 11]. Furthermore, each station uses strictly panel interview-style questions (i. e. no standardized patients) and assesses four constructs: one station-specific construct (‘self-reflection’, ‘ethical decision-making’, ‘collaboration’, or ‘values’), ‘maturity’, ‘communication and interpersonal skills’, and ‘caring and conscientiousness’ [5]. These constructs are scored using a 7-point Likert scale and summed to give a total station score [11, 12].

Unfortunately, it is difficult to confidently state that the MPI meets its goal of being as effective and more feasible than the MMI due to limited literature published about this format. Thus, this paper aims to: (1) critique validity evidence for the MPI to uncover potential shortcomings, (2) propose modifications to the MPI based on the critique, and (3) suggest research initiatives to improve understanding of the MPI.

## The critique

I used Kane’s validity framework to critique evidence for the MPI because it considers support for assumptions made at four levels of inference: scoring, generalization, extrapolation, and implication [13–18]. Based on assumptions made at each level, assessors infer a candidate’s ability, which ultimately informs their decisions about the candidate’s admission to medical school [13–18]. Therefore, assumptions must be scrutinized against evidence from the literature to ensure that inferences drawn from the MPI are trustworthy and defensible [13–18]. This is especially important since students accepted to medical school eventually practice medicine in the community and thus greatly influence the health of the general public [9]. The following four sections will examine the evidence for each level of inference.

### The ‘Scoring’ inference

Scoring involves assigning scores to observations; for the MPI, this refers to interviewers assigning Likert ratings based upon observing candidates within a station [13–18]. At this level, two assumptions require support: (1) evaluators consistently assign scores that appropriately reflect the candidates’ abilities, and (2) the tool appropriately measures constructs of interest.

For the first assumption, evaluators should be trained in using the assessment tool [13–18]. MPI interviewers are trained through mandatory online sessions; optional in-person sessions offer MPI demonstrations that are recorded for future reference [5]. Since in-person sessions are voluntary, they are poorly attended [12]. This causes evaluators to miss the chance to engage in observational learning, which can cultivate essential nontechnical skills—like decision-making—for accurately rating candidates [19]. Moreover, observing errors in rating applicants and how to overcome them is valuable external feedback that evaluators can use to improve their rating abilities and recognize erroneous tendencies [20]. Without such insight, evaluators become more susceptible to halo error, which is when their fixation on a *single* construct leads them to rate *all *constructs similarly [12]. As a result, evaluators assign scores that do not accurately represent what they observed, jeopardizing the scoring inference. Supporting the presence of halo error are high correlations (r = 0.70–0.86) between the four Likert ratings given in a single station (‘*within*-station correlations’), as these suggest that evaluators’ overall impressions of one construct may influence scoring of all four [12]. Such correlations are present at all four stations.

For the second assumption, it is important to note that, in addition to pre-determined questions, evaluators can devise questions during interviews [5]. Since the presentation questions can greatly impact how they are answered, they must be constructed such that candidates understand what is being asked; this ensures that differences in scores are attributable to differences in ability. Although specific questions cannot be obtained and scrutinized, Hanson et al. (2016) quantified the degree to which questions influence scoring using a generalizability (G) study [5]. They found that only 0.011% of the variation in scoring—a negligible amount—is due to the questions themselves, regardless of whether they were pre-determined or impromptu [5].

### The ‘Generalization’ inference

Generalization involves making overall judgements about test performance based on assigned scores [13–18]. In order for scores to reflect test performance, components chosen for a given test (e. g. evaluators, stations, questions, etc.) must be representative of all components that could have theoretically been chosen [13–18]. As such, two assumptions require support: (1) assessment content appropriately and adequately samples relevant topics and constructs, and (2) the assessment is highly likely to assign similar scores on subsequent tests that use entirely new components [13–18].

For the first assumption, it is important to ensure the assessment sufficiently covers constructs it aims to assess; one strategy for this is blueprinting, or mapping assessment content onto the constructs being assessed [21]. This seems to have been done for the MPI by adapting themes from the University of Toronto’s Leadership Education and Development program (LEAD)—a leadership program that also uses the MPI. LEAD used the following themes for blueprinting: (1) self-reflection and personal insight, (2) bandwidth and adaptability, (3) ability to work in teams, and (4) vision and expectations of [the program] [11]. Aside from exchanging ‘bandwidth and adaptability’ for ‘ethical decision-making’, these constructs directly translate to those assessed by the University of Toronto’s medical program. Since LEAD is comprised of medical students, using these same constructs to screen medical school applicants is appropriate. After reworking LEAD constructs for the medical school MPI, questions are developed for evaluators [12]. Although it seems that the medical school MPI was blueprinted, there is no explicit confirmation or description of this in the literature. This is problematic because if MPI content does not reflect the constructs it claims to assess, evaluators may inaccurately judge candidates’ performance or inadvertently assess different constructs.

For the second assumption, the MPI must have a high inter-interview reliability to show that it can discriminate consistently between candidates [5]. However, inter-interview reliability is just 0.56 for the MPI, which is below accepted standards (0.70–0.90) and supports only moderate confidence for distinguishing between candidates consistently. One potential cause for this is construct-irrelevant variance—systematic error in assessment data due to factors unrelated to constructs of interest [21, 22]. This means that factors *other than* the applicant’s competence in the assessed constructs influence their scores, compromising accurate judgement of their performance using these scores. The G study by Hanson et al. (2016) quantifies the influence of construct-irrelevant variance on the MPI [5]. Their results show that 52% of variation in scores is from a combined effect of the station and applicant; 18% is from a combined effect of the station, applicant, and items; and only 17% is from true differences between applicants [5]. These percentages show that scores assigned to candidates are significantly influenced by situational factors, suggesting context specificity [2, 6]. Further support for context specificity is within-station correlations between the four constructs (r = 0.70–0.86); scores for constructs within a station are highly related, despite being inherently different [12]. Additionally, low correlations (r = 0.06–0.27) between common constructs *across* stations (‘*between*-station correlations’) suggest that there are not enough MPI stations to sufficiently sample the assessed constructs and overcome context specificity; between-station correlations are high when constructs are aptly sampled [12]. Therefore, since situational influences are dynamic and change with repeated assessments, the MPI is unlikely to score similarly on subsequent administrations.

### The ‘Extrapolation’ inference

Extrapolation involves judging whether test performance reflects real-world performance [13–18]. For the MPI, this means inferring that candidates will succeed academically and non-academically in medical school (i. e. real-world performance) based on success during the interview (i. e. test performance). At this level, the assumption requiring support is that the chosen comparator metrics represent medical school performance [13–18].

To support this assumption, Hanson et al. (2016) examined the MPI’s predictive validity against performance on a first-year objective structured clinical examination (OSCE)—a scenario-based assessment—that tests physical examination and communication skills [5]. The authors used these scores as comparator metrics because this OSCE is encountered in medical school. The results show that MPI total scores are moderately correlated with physical examination skills (r = 0.24), communications skills (r = 0.33), and total OSCE scores (r = 0.30) [5]. These associations suggest that the MPI shows promise in selecting candidates who will perform well in first-year medical school, supporting the extrapolation inference. However, one shortcoming is that Medical College Admission Test (MCAT) scores and grade point averages (GPA) were not used as additional comparators, despite their ability to modestly, yet significantly, predict medical school and licensing examination performances [12, 23–25]. Nevertheless, there is still some support for this assumption through predictive validity evidence from the OSCE metric.

### The ‘Implication’ inference

Implication involves considering consequences of a decision on all stakeholders involved [13–18]. For the MPI, this means considering consequences of medical admissions decisions on the candidates, medical program, medical profession, and society. The assumption requiring support is that the MPI will lead to more positive outcomes for these stakeholders.

To support this assumption, it is important to consider benefits for each stakeholder. For candidates, medical school acceptances provide positive emotional reactions; rejections would cause negative emotional reactions and loss of application fees. Furthermore, the MPI benefits candidates by increasing the number of applicants that the University of Toronto can interview, resulting in more interview invites being sent [5]. For the medical program, the MPI requires less interviewers than the panel interviews, reducing burden on the University’s resources [5]. Specifically, the MPI allowed just 160 evaluators to interview 600 candidates; the panel interview required 290 interviewers for 587 candidates [5]. Moreover, since MPI scores are related to first-year OSCE performance, accepted candidates are more likely to succeed academically, reflecting positively on the program. For the profession and society, reduced error rates in practice are strong evidence. However, no data show that those accepted through the MPI commit less errors than those accepted through other formats—a gap in the literature. Nonetheless, there is still some support for this assumption as the MPI benefits candidates and the medical program.

## Proposed modifications to the MPI

Based on the critique using Kane’s framework, ‘Scoring’ and ‘Generalization’ have the least support. To bolster these inferences, two changes to the MPI are recommended: (1) in-person evaluator training should become mandatory, and (2) the number of stations should be doubled, with the additional four being blueprinted to pre-existing constructs.

Making in-person training mandatory will likely increase their attendance rates to 95%, as seen in the online sessions [5]. With this increase, more evaluators will learn through observation to develop the decision-making skills needed to appropriately rate candidates while improving their self-awareness of halo error [19–24]. The drawback of mandatory in-person training is that it requires evaluators—medical faculty, residents, and fourth-year medical students—to meet at a common time and place. Given their busy schedules, they may be unable or unwilling to attend. However, the high-stakes of medical admissions makes it crucial to sufficiently train evaluators in rating protocols such that they accurately score candidates. To increase buy-in, it may be worth incentivizing evaluators (e. g. offering credits to fourth-year medical students).

According to the decision (D) study by Hanson et al. (2016), having eight MPI stations increases inter-interview reliability from 0.56 to above 0.70—within accepted limits [5]. The four new stations must also be blueprinted onto constructs assessed in the original MPI to ensure apt sampling of each construct [12]. This would decrease context specificity and construct-irrelevant variance such that a greater variance in scores would be from true differences between candidates. Through these changes, there can be more confidence in the MPI’s ability to consistently distinguish between candidates [5]. In terms of feasibility, having eight stations would require more resources (e. g. rooms, interviewers, etc.), which begs the question: why not use the MMI? The MMI requires standardized patients, scenarios, and enough rooms for 10 stations [5, 8–10]. However, an eight-station MPI does not need standardized patients and scenarios and needs two less stations than the MMI, making it more feasible than an MMI and comparably reliable.

## Suggested research initiatives

The critique revealed several knowledge gaps regarding the MPI. For ‘Generalization’, the blueprinting process is unclear. This is problematic because even if the MPI *was* formally blueprinted, the sparse literature discussing this essential aspect of development casts doubt on its validity. Thus, one future initiative is to formally blueprint each station such that every question maps onto the four pertinent constructs.

For ‘Extrapolation’, while the MPI showed predictive validity for first-year OSCE scores, no such analyses were conducted with other comparator metrics (e. g. MCAT, GPA) [12]. Taking this one step further, researchers can examine the MPI’s predictive validity for licensing examination scores as was done for the MMI [26]. Such analyses for the MPI would enable extrapolations about real-world performance to be made more confidently.

Finally, the ‘Implication’ section revealed a lack of data regarding error rates of physicians accepted to medical school from the MPI. Collecting such data through longitudinal studies would provide insight into consequences of using the MPI—in particular, the benefits for the medical profession and society. Indeed, exploring such real-world implications can provide evidence that justifies using the MPI at other institutions.

## Conclusion

There are currently no publications that synthesize and critique evidence for the MPI to the degree explored in this paper. While the MPI significantly improves upon the panel interview, it is currently not as effective as the MMI. Using Kane’s validity framework, the MPI was critiqued and its shortcomings were revealed. Specifically, the ‘Scoring’ section showed that rater training can be optimized by making in-person sessions mandatory; the ‘Generalization’ section revealed that construct-irrelevant variance and context specificity can be minimized by having eight stations instead of four and blueprinting questions in these stations to pre-existing constructs; the ‘Extrapolation’ section revealed that associations between the MPI and other metrics (e. g. MCAT, GPA) should be explored to increase predictive validity evidence; and the ‘Implication’ section revealed benefits of the MPI for candidates and the medical program.

For institutions using formats other than the MPI (i. e. panel interview, MMI), this article describes an attractive alternative to consider that is feasible and highly effective for screening applicants. Moreover, for institutions using the MPI, this article offers potential revisions that can improve the process by making it as reliable as the MMI while remaining logistically sound. Finally, researchers are encouraged to explore the suggested initiatives to build evidence to support each level of inference in Kane’s framework, thereby making a stronger case for the MPI.
